# Association of urinary heavy metals with chronic pain depression co-morbidity: based on NHANES

**DOI:** 10.3389/fpubh.2025.1718605

**Published:** 2025-11-28

**Authors:** Long Li, Junwei Liu, Li Ding, Yongli Wu

**Affiliations:** 1 Orthopaedics Department of Traditional Chinese Medicine, General Hospital of Ningxia Medical University, Yinchuan, Ningxia Hui Autonomous Region, China; 2 Key Laboratory of Ningxia Ethnomedicine Modernization, Ministry of Education, Ningxia Medical University, Yinchuan, Ningxia Hui Autonomous Region, China

**Keywords:** urinary heavy metals, chronic pain-depression co-morbidity, nutrition examination survey, restricted cubic spline, Bayesian kernel-machine regression

## Abstract

**Introduction:**

Accumulating evidence indicates that environmental exposure to toxic metals poses significant risks to human health, yet limited evidence exists regarding the neuropsychiatric effects of mixed metal exposure, particularly in the context of pain-depression co-morbidity (PDC).

**Methods:**

To address this gap, we analyzed data from the National Health and Nutrition Examination Survey (NHANES) to evaluate individual and synergistic associations between 12 urinary metals and PDC risk using multi-variable logistic regression, restricted cubic spline (RCS) analysis, and Bayesian kernel machine regression (BKMR).

**Results:**

This cross-sectional study of 1,202 participants identified a PDC prevalence of 12.4% (*n* = 149). Fully adjusted logistic regression identified four metals with significant positive associations with PDC: cadmium (Cd) (odds ratio [OR] = 1.293, 95% confidence interval [CI]: 1.116–1.500, *p* = 0.001), cobalt (Co) (OR = 1.300, 95% CI: 1.089–1.548, *p* = 0.003), thallium (Tl) (OR = 1.243, 95% CI: 1.042–1.478, *p* = 0.015), and tungsten (Tu) (OR = 1.158, 95% CI: 1.017–1.317, *p* = 0.026). Linear regression confirmed these associations, with uranium (Ur) showing an additional significant link (*β* = 0.039, *p* = 0.033). RCS analysis revealed a nonlinear relationship for Tu (*p* = 0.036). BKMR modeling demonstrated a positive overall effect of mixed metal exposure, with Tu (posterior inclusion probability [PIP] = 0.91), Tl (PIP = 0.88), Cd (PIP = 0.85), and Co (PIP = 0.82) as the predominant contributors. In single-metal exposure profiles, PDC scores increased dose-dependently with rising concentrations of Tu, Tl, and Cd when co-exposures were fixed at their 25th, 50th, and 75th percentiles. These findings identify urinary Tu, Tl, Cd, and Co as critical biomarkers for PDC risk, revealing their non-monotonic and dose-responsive neurotoxic effects. The BKMR-derived synergy among metal mixtures underscores the necessity of evaluating environmental exposures holistically rather than in isolation.

**Discussion:**

These results advance our understanding of metal-induced neuropsychiatric pathophysiology and support the integration of metal exposure screening into clinical assessments of affective-somatic disorders.

## 1 Introduction

Pain, a multidimensional sensory and emotional experience associated with actual or potential tissue damage, represents a critical public health challenge with profound physiological and psychological consequences (1). Chronic pain – clinically defined as persistent or recurrent discomfort exceeding three months – affects over 30% of the global population, imposing substantial socioeconomic burdens through functional impairment and reduced quality of life (2). Depression, characterized by persistent anhedonia and affective dysregulation, frequently coexists with chronic pain, establishing a bidirectional pathophysiological interplay that complicates clinical management (3–5). Epidemiologic data reveal that 52% of chronic pain patients exhibit depressive symptoms, with prevalence escalating with multisite pain localization (6). Notably, migraineurs demonstrate a 3.6-fold elevated risk of incident depression compared to the general population (7), while 65% of depressed individuals report concurrent pain syndromes involving musculoskeletal, visceral, or neuropathic components (6). This pain-depression comorbidity (PDC) exhibits high relapse rates (>50%) and therapeutic resistance, often persisting through remission phases and requiring multimodal intervention strategies (8).

Emerging evidence implicates environmental toxicants, particularly bioaccumulative heavy metals, as modifiable risk factors for PDC pathogenesis (9–11). Ubiquitous environmental contaminants such as mercury, lead, and cadmium disrupt neuroendocrine-immune networks through multifaceted mechanisms, including direct neurotoxicity mediated by blood–brain barrier penetration and mitochondrial dysfunction, epigenetic modulation of pain-associated ion channels and monoaminergic pathways, as well as the induction of systemic oxidative stress and neuroinflammation (12). Clinical observations demonstrate that chronic mercury exposure correlates with idiopathic myalgia and cephalalgia (13), while lead intoxication manifests as chronic abdominal pain coupled with affective dysregulation (14). Cadmium bioaccumulation induces both nephrotoxicity and characteristic “aching pain” syndromes through musculoskeletal redox imbalance (15, 16).

However, most prior investigations have predominantly focused on single-metal effects, which stands in stark contrast to the reality of ubiquitous co-exposure to multiple metals from sources like industrial emissions and contaminated groundwater. This narrow focus creates critical interpretative limitations. Firstly, it fails to account for the “mixture effect,” where the combined toxicity of concurrent exposures may differ from the sum of individual parts, potentially leading to an underestimation of the true health risk. More importantly, single-metal models fail to capture the complex interactive effects inherent to metal mixtures, such as synergy, where co-exposure to cadmium and lead results in greater renal toxicity than would be expected (17), and antagonism, where zinc mitigates cadmium toxicity (18). For example, studies on other health outcomes such as a study on cardiovascular risk have shown that the overall risk is often driven by specific metal clusters, with the effect of one metal being dependent on the concentrations of others (19). Consequently, conclusions from single-metal studies may not reflect the true risk profile, potentially overlooking key drivers or misattributing effects. Given this critical knowledge gap and the notable absence of studies specifically investigating the association between mixed metal exposure and chronic PDC, an in-depth exploration is warranted. Our study aims to address this by employing multiple statistical models, including BKMR, to holistically assess both the individual and combined effects of metal mixtures on PDC, thereby providing valuable etiological clues and informing public health prevention and clinical management strategies for this comorbidity.

## 2 Methods

### 2.1 Data and samples

**Figure 1:**
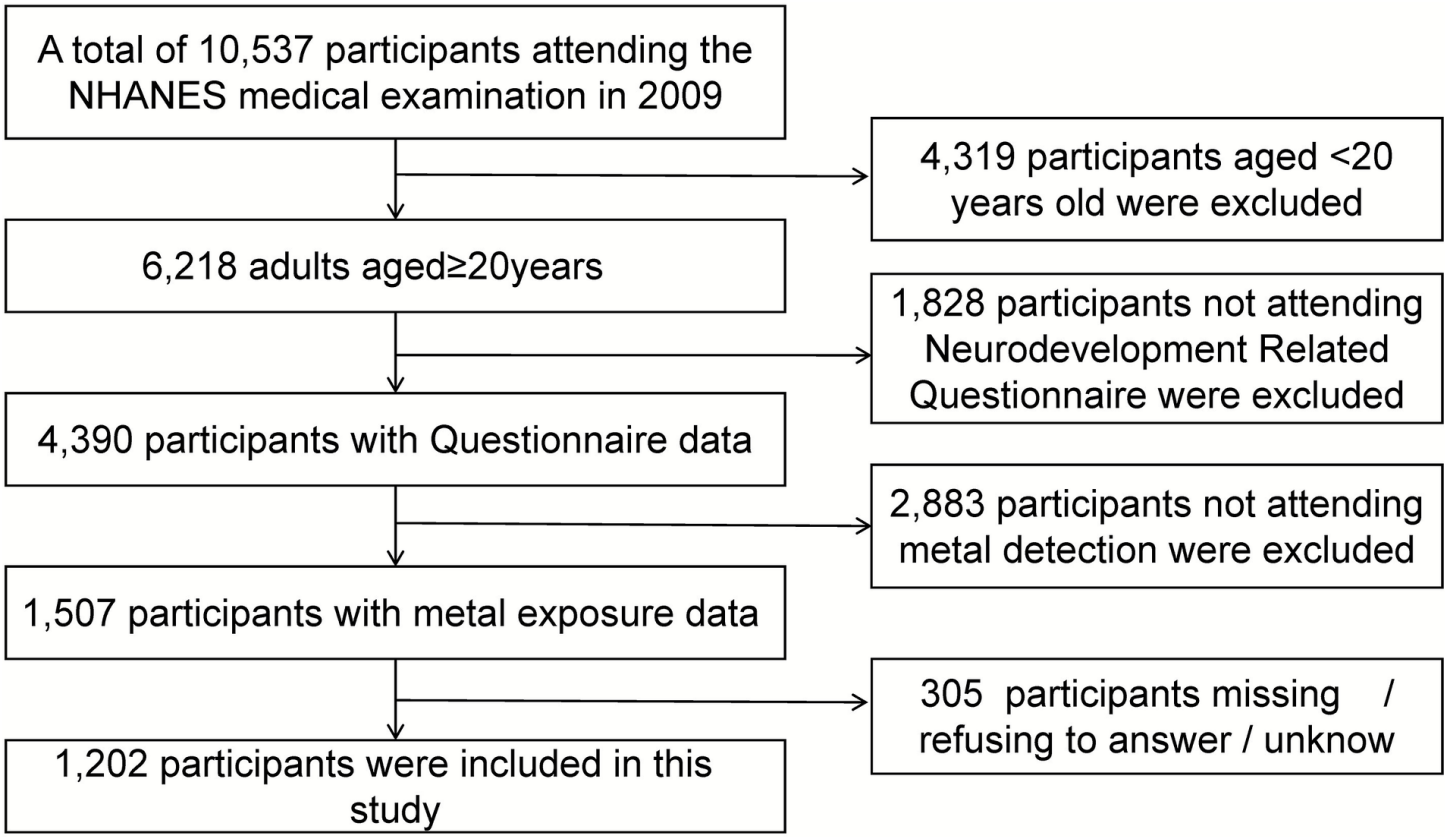
Sample screening flowchart.

The National Health and Nutrition Examination Survey (NHANES), is a population-based, cross-sectional survey designed to collect information on the health and nutritional status of adults and children in the United States. The program surveys a nationally representative sample of 5,000 people located in every county in the country each year. The study sample is drawn primarily from the 2009–2010 cycle of NHANES. The cycle uses a stratified multistage probabilistic clustering design to obtain a representative sample of the civilian, non-institutionalized U.S. population conducted by the Centers for Disease Control and Prevention (CDC). Participants were 10,537 U.S. population members who participated in the NHANES survey and who were administered urine heavy metal measurements and pain-depression-related questionnaires. Inclusion exclusion criteria are shown in Figure 1. The final analytic sample consisted of 1,202 samples. The NCHS Institutional Review Board approved the NHANES protocol, and all study participants provided written informed consent.

### 2.2 Measurement of exposure

NHANES investigators collected urine specimens from subjects, which were processed, stored, and transported to the Environmental Health Laboratory Sciences Division of the National Center for Environmental Health at the Centers for Disease Control and Prevention for analysis. Inductively coupled plasma mass spectrometry (ICP-MS) was used to measure the following 12 elements in the urine: beryllium (Be), cobalt (Co), molybdenum (Mo), cadmium (Cd), stibium (Sb), cesium (Cs), barium (Ba), tungsten (Tu), platinum (Pt), thallium (TI), lead (Pb), and uranium (U), with the resultant value for the below-limit-of-detection (LOD) assay being recorded as LOD divided by √2. To avoid biased results due to urine dilution, creatinine-calibrated concentration values were used in subsequent analyses.

### 2.3 Measurement of outcome

Pain was measured using the Arthritis ARQ_F questionnaire, which includes the following items: “ARQ020A – Had Neck Pain for 6 Weeks,” “ARQ020B – Had Upper Back Pain for 6 Weeks,” “ARQ020C – Had Mid Back Pain for 6 Weeks,” “ARQ020D – Had Low Back Pain for 6 Weeks,” “ARQ020E – Had Buttocks Pain for 6 Weeks,” “ARQ020F – Had Hip Pain for 6 Weeks,” and “ARQ020G – Had Rib Cage Pain.”

Depressive symptoms were assessed with the Patient Health Questionnaire (PHQ-9), a 9-item screening tool that evaluates the frequency of depressive symptoms over the past 2 weeks. The final item of the PHQ-9 assesses the overall impairment caused by depressive symptoms. Each response category—"not at all,” “a few days,” “more than half the days,” and “almost every day”—is scored on a scale of 0 to 3. The total score of the PHQ-9 ranges from 0 to 27.

We constructed two complementary variables for pain-depression comorbidity (PDC) analysis. A binary comorbidity variable (primary outcome): Defined as the co-occurrence of chronic pain (pain score > 0) and clinically relevant depression (PHQ-9 score ≥ 5, a validated threshold widely used in epidemiological studies) (20). A standardized composite score (secondary outcome): To explore the dimensionality of comorbidity severity, we first standardized the pain score (range 0–2) and PHQ-9 score (range 0–27) to a 0–1 scale (eliminating biases from differing scale ranges) before summing them.

### 2.4 Covariates

The following covariates were included in this study: Age (continuous), Gender (male or female), Race/ethnicity (Mexican American, Other Hispanic, Non-Hispanic White, Non-Hispanic Black, Other Race/Multiracial), Country of birth (born in the 50 U.S. or Washington, D.C., born in Mexico, born elsewhere), marital status (Married, Widowed, Divorced, Separated, Never married, Living with partner), whether or not they drink alcohol (Yes, No), whether or not they smoke (Yes, No), Level of work activity (None, Moderate and Vigorous), Level of recreational activities (None, Moderate and Vigorous), Education level (Less Than 9th Grade, 9–11th Grade, High School Grad/GED or Equivalent, Some College or AA degree, College Graduate or above), Family income-to-poverty ratio, sleep duration, body mass index (BMI) and other chronic disease (Diabetes, hypertension) (21). Subjects used a self-administered questionnaire to collect sociodemographic and lifestyle information.

### 2.5 Statistical analysis

Considering a complex sample design for NHANES, R (4. 3.0) and StataMP.15 software were used for data analysis. Continuous variables were compared using the Mann–Whitney U test since normality assumptions were not met, while categorical variables were analyzed using the chi-square test. Heavy metal concentrations for each category were log-transformed (continuous) in urine. The relationship between single metals and PDC was estimated by building logistic regression in three models: model 1 analyzed only the relationship between single metals and PDC, model 1 adjusted for basic demographic information such as age and gender, and model 3 was based on model 2 incorporating lifestyle factors such as smoking and alcohol consumption. Results are shown as odds ratios (OR) and corresponding 95% confidence intervals (CI) (OR, (95% CI)). Restricted cubic spline plots were used to assess the nonlinear relationship between urinary metals and PDC. Spearman correlation analysis was used to assess correlations between log-transformed metals in urine.

Given the potentially nonlinear and noncumulative dose–response relationships between metals, BKMR was used to assess the joint effect of various metalloids on the risk of PDC. The method integrates Bayesian and statistical learning methods to estimate nonlinearities and/or interactions in exposure-outcome associations. BKMR is characterized by the flexibility of exposure-response function modeling and helps to visualize the effects of single or combined exposures. We performed 50,000 iterations to assess the joint effect of metal co-exposure on PDC risk using the BKMR package in R software. Simultaneously, exposure-response and dose–response functions for single metal and PDC risk were obtained.

## 3 Results

### 3.1 Study population characteristics

The overall characteristics of the study population are presented in Table 1. This cross-sectional study of 1,202 participants identified a PDC prevalence of 12.4% (*n* = 149). Multivariable analyses revealed significant disparities across demographic, socioeconomic, and clinical domains. The PDC cohort demonstrated a pronounced female predominance (60.4% *vs* 44.6%) and substantially lower socioeconomic status, as evidenced by reduced family poverty-income ratios (median PIR: 1.4 *vs* 2.2,). Health behavior assessment identified significantly compromised sleep patterns among PDC participants (median sleep duration: 6 *vs* 7 h) and markedly higher rates of physical inactivity (63.8% *vs* 46.7%). Educational disparities were particularly striking, with the PDC group exhibiting both elevated rates of limited formal education (<9th grade: 14.1% *vs* 7.7%) and substantially diminished college completion rates (8.7% *vs* 22.3%). Clinical profiles further distinguished the cohorts, with PDC participants demonstrating significantly greater chronic disease burden (40.3% *vs* 29.2%) despite lower tobacco use prevalence (51% *vs* 70.9%). These findings delineate a distinct phenotypic profile associated with pain-depression comorbidity, characterized by socioeconomic disadvantage, health behavior compromises, and enhanced multimorbidity susceptibility.

**Table 1:** Characteristics of study population by PDC (*n* = 1,202).

Variables	Total (1,202)	Stratified by gender		*P*
		No PDC (1053)	PDC (149)	
Age (*M* (*P_25_*, *P_75_*))	44.0 (32.0, 55.0)	43.0 (31.0, 55.0)	44.0 (35.0, 56.0)	0.35
Family PIR (*M* (*P_25_*, *P_75_*))	2.1 (1.0, 4.1)	2.2 (1.1, 4.3)	1.4 (0.8, 3.0)	<0.001
BMI (*M* (*P_25_*, *P_75_*))	28.0 (24.4, 32.9)	27.9 (24.4, 32.5)	28.9 (24.2, 35.1)	0.17
Sleep time (*M* (*P_25_*, *P_75_*))	7.0 (6.0, 8.0)	7.0 (6.0, 8.0)	6.0 (5.0, 7.0)	<0.001
Gender (%)				<0.001
Male	53.4	55.4	39.6	
Female	46.6	44.6	60.4	
Race (%)				0.39
Mexican American	18.6	18.7	17.4	
Other Hispanic	10.9	11.2	8.7	
Non-Hispanic White	48.1	47.2	54.4	
Non-Hispanic Black	17.4	17.9	13.4	
Other	5.1	4.9	6	
Born Country (%)				0.02
US States or Washington	73.1	71.8	82.6	
Mexico	11.3	11.5	10.1	
Other Spanish Speaking Country	7.2	7.9	2.7	
Others	8.3	8.8	4.7	
Marital (%)				<0.001
Married	51.3	53.6	35.6	
Widowed	3.5	2.8	8.7	
Divorced	11.3	10.6	16.1	
Separated	5	4.6	8.1	
Never married	18.2	18.2	18.1	
Living with partner	10.6	10.3	13.4	
Whether drink (%)				0.12
No	15.5	14.8	20.1	
Yes	84.5	85.2	79.9	
Physical activity time (%)				<0.001
None	48.8	46.7	63.8	
Moderate	28	28.8	22.1	
Vigorous	23.2	24.5	14.1	
Recreation time (%)				0.6
None	54.8	55	53.7	
Moderate	21.8	21.4	24.8	
Vigorous	23.4	23.6	21.5	
Whether smoke (%)				<0.001
No	31.5	29.1	49	
Yes	68.5	70.9	51	
Education level (%)				<0.001
Less than 9th Grade	8.5	7.7	14.1	
9–11th Grade	15.3	14.5	20.8	
High School or Equivalent	25	25.1	24.2	
Some College or AA degree	30.6	30.4	32.2	
College Graduate or above	20.6	22.3	8.7	
Whether developed chronic disease (%)				<0.01
No	69.4	70.8	59.7	
Yes	30.6	29.2	40.3	

PIR, Poverty Income Ratio; BMI, Body Mass Index; M, Median; P_25_, 25th Percentile; P_75_, 75th Percentile.

### 3.2 Heavy metal concentrations and correlations

**Figure 2:**
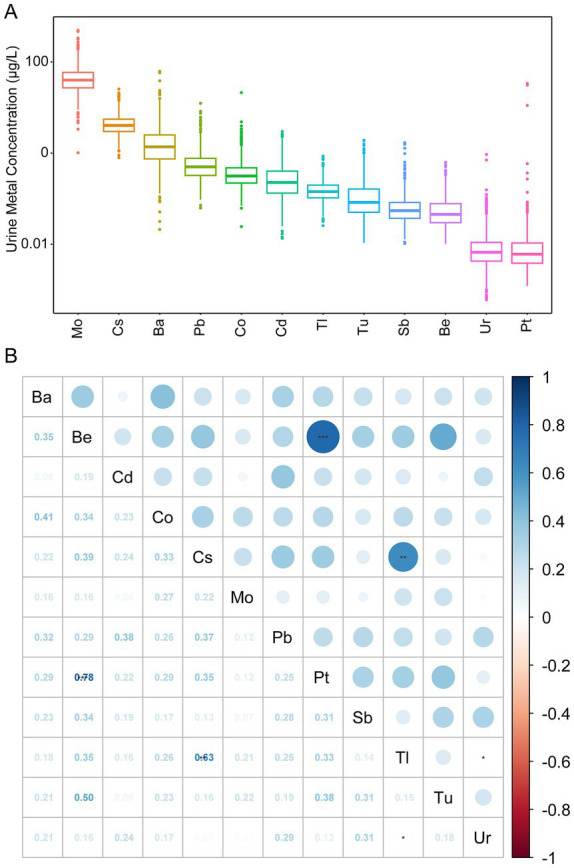
Heavy metal concentrations and correlations. **(A)** Distribution of heavy metal concentrations in NHANES, 2009–2010, with the names of the various metals in the horizontal coordinates and the concentration values of the various metals in the vertical coordinates, with a log10 transformation of the *y*-axis. **(B)** Correlations of 12 metals calculated using Spearman’s correlation method. The numbers in the lower left corner are the correlation coefficients, and the upper right part is a heat map of the correlation coefficients between metal concentrations. White color indicates zero correlation, blue color indicates positive correlation, and red color indicates negative correlation. The darker the color, the greater the correlation coefficient. Black *, ** and *** indicate *p* < 0.05, *p* < 0.01 and *p* < 0.001, respectively.

Table 2 shows the distribution of diseases and 12 metals by gender. Women had higher pain depression scores compared to men, and women had higher levels of metal exposure than men. In addition, the overall distribution of heavy metals is visualized in Figure 2A. Figure 2B is a heat map showing the correlation between the 12 heavy metals using the Spearman correlation matrix. Complex exposure profiles were observed between metal concentrations, with a roughly positive trend in paired Spearman correlations. We found high correlations between urinary Be and urinary Pt (Spearman correlation *ρ* = 0.78), and urinary Cs and urinary Tl (*ρ* = 0.63).

**Table 2:** Distribution of PDC and heavy metal concentrations (*n* = 1,202).

Metal	*P*_25_	*M*	*P*_75_	Males (Mean (SD))	Females (Mean (SD))
Ba	0.845	1.5	2.688	2.22 (4.88)	2.69 (3.45)
Be	0.0307	0.0476	0.082	0.06 (0.05)	0.09 (0.08)
Cd	0.12	0.209	0.382	0.26 (0.29)	0.37 (0.33)
Co	0.231	0.326	0.494	0.31 (0.21)	0.69 (1.47)
Cs	3.05	4.15	5.93	4.27 (2.41)	5.44 (2.87)
Mo	27.4	40	59.1	43.94 (37.19)	54.11 (39.41)
Pb	0.312	0.468	0.727	0.62 (0.67)	0.62 (0.49)
Pt	0.0039	0.00638	0.01132	0.02 (0.69)	0.06 (0.95)
Sb	0.0365	0.0541	0.0818	0.07 (0.08)	0.08 (0.07)
Tl	0.107	0.148	0.207	0.15 (0.08)	0.20 (0.12)
Tu	0.0516	0.0866	0.175	0.15 (0.22)	0.18 (0.22)
Ur	0.00413	0.00646	0.01079	0.01 (0.03)	0.01 (0.05)

PDC, Pain-Depression Comorbidity; M, Median; P_25_, 25th Percentile; P_75_, 75th Percentile; SD, Standard Deviation.

### 3.3 Associations between urinary metal concentrations and PDC

**Figure 3:**
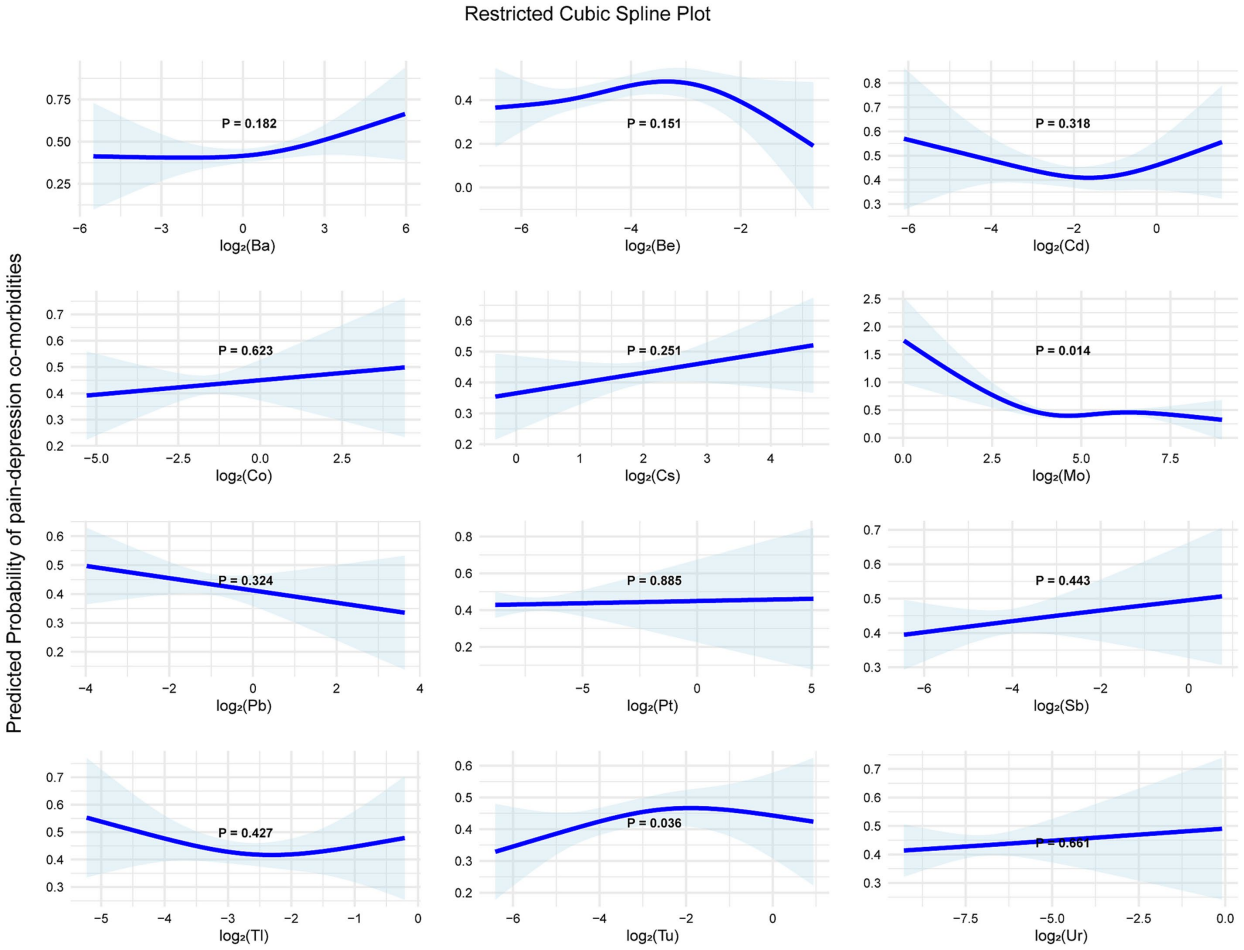
Restricted cubic spline plot. Each subplot illustrates the predicted probability of pain-depression comorbidity across the range of log_2_-transformed urinary metal concentrations, with shaded areas representing 95% confidence intervals. Models were adjusted for gender, age, race, country, education level, marital status, family income-to-poverty ratio, BMI, whether drink, physical activity time, sleep time, recreation time, whether smoke and other chronic disease. *p*-values indicate the statistical significance of nonlinearity for each metal.

This study investigated associations between urinary metal concentrations and PDC. In multiple logistic regression (Table 3), four metals were significantly associated with PDC presence after full confounder adjustment (Model 3): Cd (OR = 1.293, 95% CI: 1.116–1.500, *p* = 0.001), Co (OR = 1.300, 95% CI: 1.089–1.548, *p* = 0.003), Tl (OR = 1.243, 95% CI: 1.042–1.478, *p* = 0.015), and Tu (OR = 1.158, 95% CI: 1.017–1.317, *p* = 0.026), while Ur showed a marginal association (OR = 1.156, 95% CI: 0.985–1.350, *p* = 0.070). Consistent with the primary outcome, multiple linear regression (standardized PDC composite score) revealed five metals with significant positive associations in Model 3 (Table 4): Cd (*β* = 0.055, 95% CI: 0.024–0.087, *p* = 0.001), Co (β = 0.055, 95% CI: 0.014–0.095, *p* = 0.008), Tl (β = 0.053, 95% CI: 0.013–0.093, *p* = 0.009), Tu (β = 0.026, 95% CI: 0.002–0.055, *p* = 0.043), and Ur (β = 0.039, 95% CI: 0.003–0.075, *p* = 0.033). Restricted cubic spline analysis identified significant nonlinear relationships for Mo (*p* = 0.014) and Tu (*p* = 0.036) with PDC probability, whereas no significant nonlinearity was observed for Ba, Be, Cd, Co, Cs, Pb, Pt, Sb, Tl, and Ur (all *p* > 0.05) (Figure 3).

**Table 3:** Multiple logistic regression modeling of urine metal exposure and PDC.

Metals	Model 1		Model 2		Model 3	
	OR(95%CI)	*P*	OR(95%CI)	*P*	OR(95%CI)	*P*
Ba	1.094 (0.956, 1.253)	0.193	1.092 (0.958, 1.246)	0.188	1.1 (0.968, 1.251)	0.145
Be	1.083 (0.901, 1.298)	0.391	1.071 (0.893, 1.28)	0.453	1.087 (0.919, 1.28)	0.326
Cd	0.963 (0.785, 1.18)	0.715	1.109 (0.919, 1.339)	0.279	1.293 (1.116, 1.5)	0.001
Co	1.204 (0.97, 1.487)	0.088	1.221 (0.991, 1.498)	0.058	1.3 (1.089, 1.548)	0.003
Cs	0.923 (0.693, 1.227)	0.583	0.92 (0.693, 1.218)	0.561	0.904 (0.703, 1.158)	0.425
Mo	1.06 (0.865, 1.301)	0.575	1.047 (0.858, 1.279)	0.653	1.047 (0.866, 1.267)	0.636
Pb	0.833 (0.664, 1.038)	0.109	0.873 (0.703, 1.078)	0.212	0.978 (0.816, 1.168)	0.810
Pt	1.031 (0.876, 1.193)	0.692	1.03 (0.878, 1.187)	0.698	1.043 (0.898, 1.191)	0.556
Sb	0.809 (0.616, 1.058)	0.123	0.818 (0.629, 1.062)	0.133	0.8 (0.628, 1.016)	0.069
Tl	1.078 (0.882, 1.309)	0.453	1.115 (0.918, 1.346)	0.263	1.243 (1.042, 1.478)	0.015
Tu	1.13 (0.981, 1.301)	0.089	1.147 (0.998, 1.317)	0.052	1.158 (1.017, 1.317)	0.026
Ur	1.025 (0.856, 1.217)	0.784	1.047 (0.879, 1.239)	0.595	1.156 (0.985, 1.35)	0.070

Model1: Unadjusted mode l; Model 2: Adjusted by gender, age, race, country, education level, marital status, family income-to-poverty ratio, BMI; Model 3: Adjusted by gender, age, race, country, education level, marital status, family income-to-poverty ratio, BMI, whether drink, physical activity time, sleep time, recreation time, whether smoke and other chronic disease. *P* < 0.05 is considered statistically different.

**Table 4:** Multiple linear regression modeling of urine metal exposure and PDC.

Metals	Model 1		Model 2		Model 3	
	*β*(95%CI)	*P*	*β*(95%CI)	*P*	*β*(95%CI)	*P*
Ba	0.021 (−0.006, 0.048)	0.133	0.02 (−0.008, 0.047)	0.158	0.026 (−0.002, 0.053)	0.071
Be	0.015 (−0.022, 0.052)	0.431	0.011 (−0.026, 0.048)	0.562	0.022 (−0.014, 0.059)	0.228
Cd	−0.006 (−0.046, 0.033)	0.753	0.012 (−0.026, 0.051)	0.525	0.055 (0.024, 0.087)	0.001
Co	0.011 (−0.033, 0.055)	0.622	0.017 (−0.028, 0.061)	0.463	0.055 (0.014, 0.095)	0.008
Cs	0.033 (−0.024, 0.09)	0.251	0.029 (−0.028, 0.087)	0.319	0.028 (−0.025, 0.082)	0.297
Mo	−0.012 (−0.053, 0.028)	0.556	−0.014 (−0.055, 0.027)	0.516	−0.009 (−0.049, 0.032)	0.684
Pb	−0.021 (−0.063, 0.021)	0.323	−0.016 (−0.058, 0.026)	0.462	0.006 (−0.032, 0.045)	0.757
Pt	0.002 (−0.03, 0.034)	0.882	0.001 (−0.032, 0.033)	0.967	0.01 (−0.022, 0.042)	0.546
Sb	−0.018 (−0.071, 0.035)	0.509	−0.015 (−0.068, 0.038)	0.584	−0.015 (−0.066, 0.036)	0.561
Tl	0.016 (−0.024, 0.055)	0.434	0.025 (−0.015, 0.065)	0.213	0.053 (0.013, 0.093)	0.009
Tu	0.019 (−0.01, 0.047)	0.199	0.02 (−0.008, 0.049)	0.163	0.026 (0.002, 0.055)	0.043
Ur	0.008 (−0.027, 0.044)	0.651	0.011 (−0.025, 0.047)	0.542	0.039 (0.003, 0.075)	0.033

Model 1: Unadjusted mode l; Model 2: Adjusted by gender, age, race, country, education level, marital status, family income-to-poverty ratio, BMI; Model 3: Adjusted by gender, age, race, country, education level, marital status, family income-to-poverty ratio, BMI, whether drink, physical activity time, sleep time, recreation time, whether smoke and other chronic disease. *P* < 0.05 is considered statistically different.

### 3.4 BKMR analysis between urinary metal and PDC

**Figure 4:**
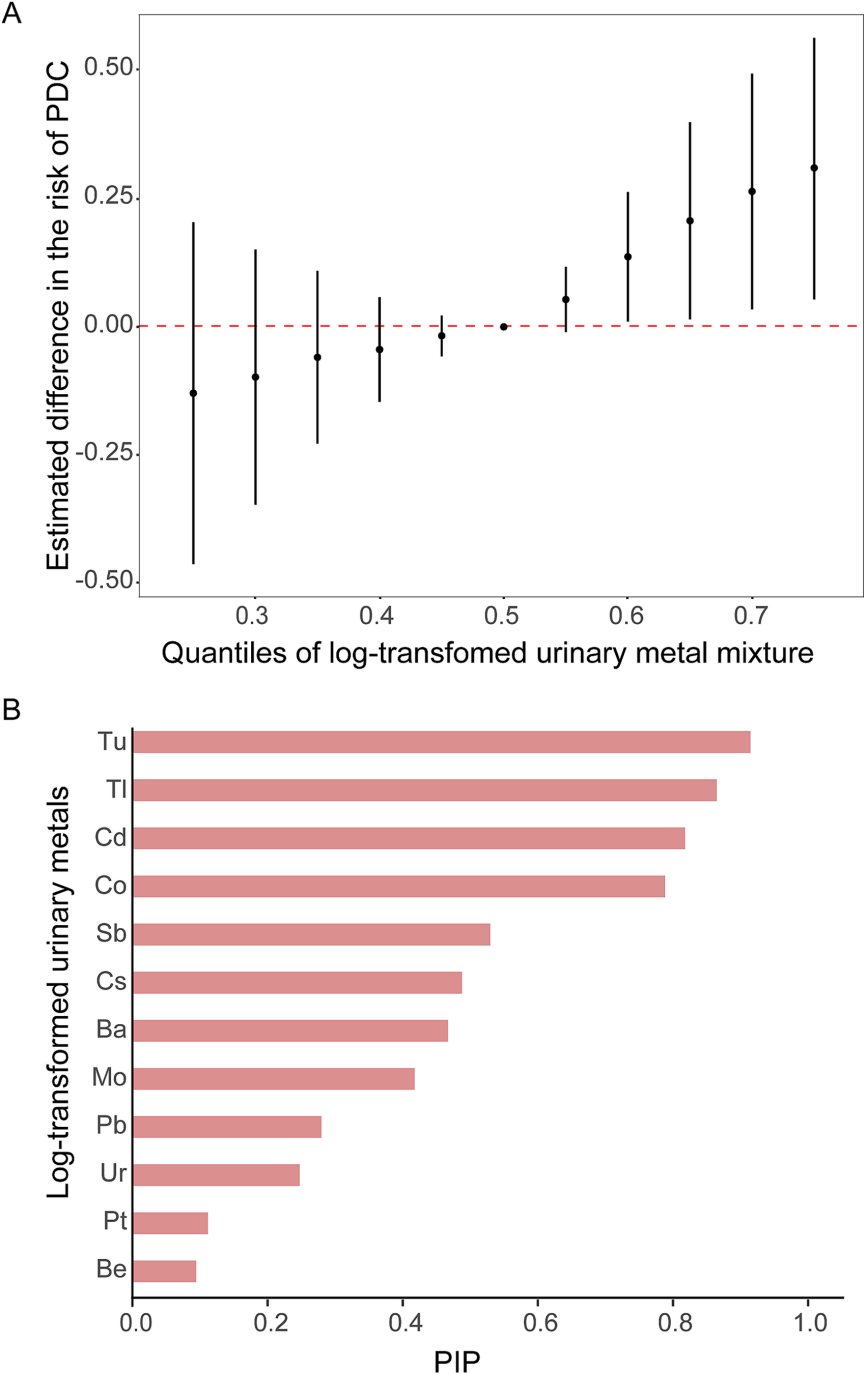
Mixed analysis of metals and PIP of Metals. **(A)** Mixed analysis of metals. Models were adjusted for gender, age, race, country, education level, marital status, family income-to-poverty ratio, BMI, whether drink, physical activity time, sleep time, recreation time, whether smoke and other chronic disease. *Y*-axis indicates the estimated difference in *z*-scores when each class of metals is fixed at a specific quartile (ranging from 0.25 to 0.75), compared to when that class of metal is at the 50th percentile. Dots indicate estimates and the black vertical line indicates the 95% CI. **(B)** PIP of Metals.

**Figure 5:**
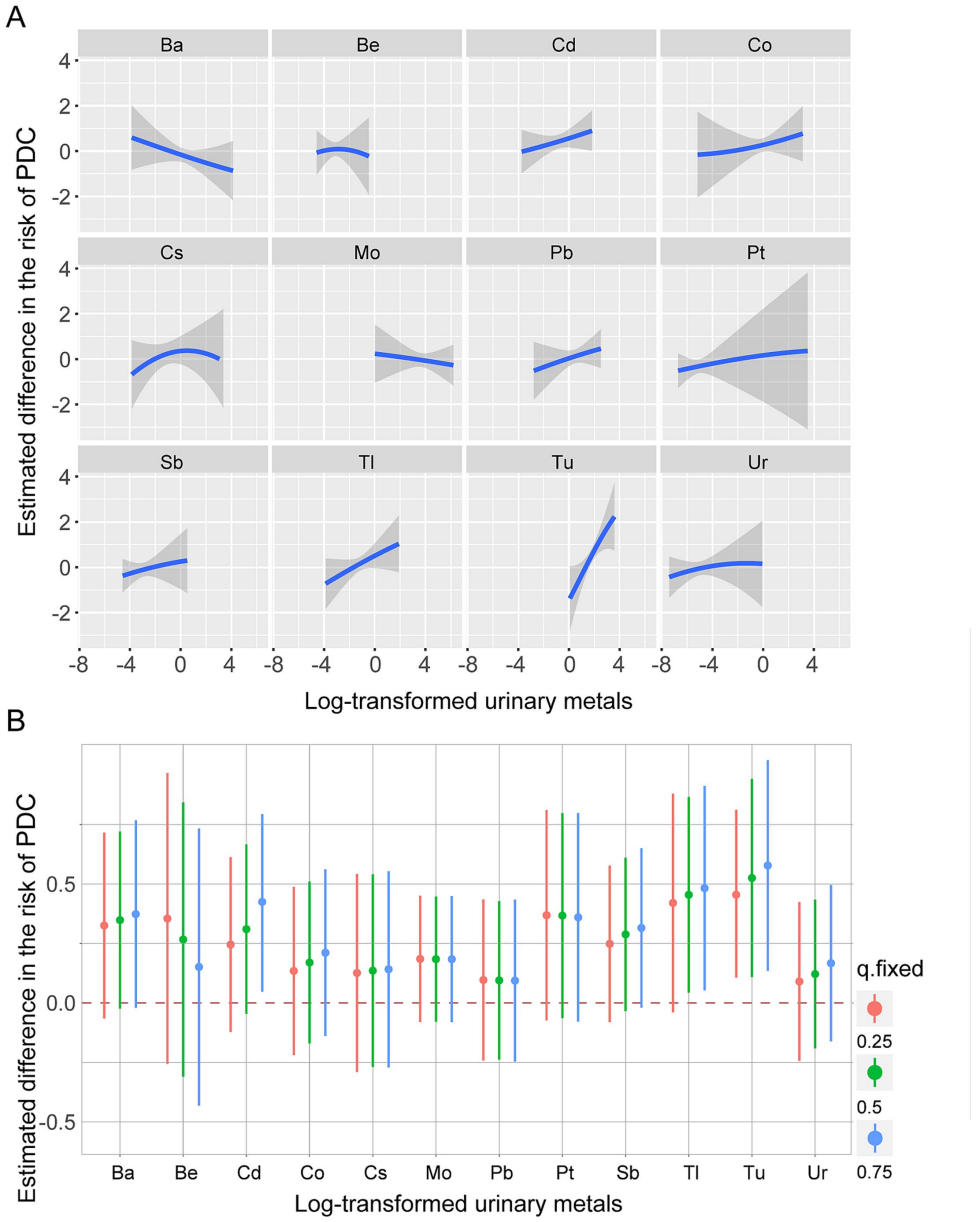
Single exposure effects. **(A)** Univariate CR curves for a single metal in PDC when other metals were fixed at the median. *Y*-axis indicates the estimated difference between the *z*-value of the level of a specific metal and the median level when all other metals are fixed at the median. **(B)** Effect of single metal on PDC. Difference in the association of single metal with PDC when itself is located at the 75th and 25th percentiles when the remaining 11 variables are fixed at the 25th, 50th, and 75th percentiles, respectively.

Considering the correlations and nonlinear relationships among urinary metals, the BKMR model was further employed to evaluate the relationship between mixed urinary metal exposure and PDC. Significant positive associations between mixed metal exposure and PDC were observed, with exposure-response curves showing monotonic risk escalation across quantiles (Figure 4A), consistent with single-metal regression results. Posterior inclusion probabilities (PIPs) identified the predominant contributors to PDC risk as Tu (PIP = 0.91), Tl (PIP = 0.88), Cd (PIP = 0.85), and Co (PIP = 0.82) – findings highly concordant with logistic and linear regression outcomes (Figure 4B). Concentration-response and single-metal marginal effect analyses (Figures 5A,B) further demonstrated that Tu, Tl, Cd, and Co exhibited dose-dependent positive associations with PDC risk, with Tu, Tl and Cd acting as independent risk amplifiers across all co-exposure scenarios. Collectively, these findings align with single-metal regression results, highlighting Tu, Tl, Cd, and Co as the key metals driving PDC risk in the mixture.

## 4 Discussion

Utilizing data from the NHANES 2009–2010 database, this study comprehensively integrated linear/nonlinear analytical approaches and single/mixed-exposure analysis methods to systematically investigate the association between urinary metal exposure and PDC. The findings demonstrated that Tu, Tl, Cd and Co exert significant linear and nonlinear effects on PDC, with BKMR further confirming their positive associations and the cumulative impact of mixed metal co-exposure. These results not only advance our understanding of environmental metal exposure as a risk factor for PDC but also highlight the complex, multi-pathway mechanisms through which these metals may synergistically or independently contribute to the PDC.

This study reveals a complex relationship between urinary metal exposure and PDC, with Tu demonstrating both linear and non-linear dose–response patterns. As an environmentally persistent transition metal, Tu’s geochemical behavior challenges early assumptions of inertness. While historically considered stable in soil matrices, contemporary evidence indicates its transformation into mobile tungstate anions (WO₄^2−^) under natural conditions, enabling entry into biological systems through root uptake and trophic transfer (22–25). This environmental mobility aligns with our findings of Tu’s bioaccumulation potential in humans, evidenced by its prolonged retention in skeletal, neural, and visceral tissues despite renal clearance mechanisms (26–28). The observed association between Tu exposure and PDC pathogenesis may stem from its dual role as a pro-inflammatory mediator and structural disruptor. Experimental models demonstrate that chronic Tu exposure induces sustained neuroinflammation through microglial activation and cytokine dysregulation, a plausible mechanism for pain-depression comorbidity (29, 30). Furthermore, clinical studies documenting Tu accumulation in spinal components suggest its capacity to accelerate disc degeneration via oxidative stress pathways, potentially exacerbating chronic pain states (31). A Canadian study found that the metal Tu tends to accumulate in intervertebral discs and vertebrae, stimulating disc degeneration and increasing markers of inflammation and pain (32). Another epidemiologic study suggests that concurrent exposure to Cd, Sb, Co, Tu, and U is associated with an increased risk of anxiety (33).

It is generally recognized that Tl is a toxic heavy metal element and a nonessential trace element that can enter and enrich the body through drinking water, food, and breathing, and is potentially neurotoxic, and its compounds are mutagenic, carcinogenic, and teratogenic (34). The neurotoxic profile of Tl emerges as another critical determinant of PDC risk in our analyses. Despite urinary Tl concentrations (mean: 0.15 μg/g creatinine) remaining below acute toxicity thresholds, its chronic exposure effects warrant serious consideration. Tl’s molecular mimicry of potassium ions allows disruption of neural electrophysiology, while mitochondrial toxicity in CNS cells may underlie subclinical neuropsychiatric manifestations (35, 36). This subtle yet persistent neurotoxicity could explain the population-level association with PDC, particularly when compounded by lifetime exposure accumulation. The differential tissue tropism between these metals—Tu’s skeletal affinity versus Tl’s neural sequestration—suggests distinct yet complementary pathways in PDC development, potentially creating synergistic effects through systemic inflammation and neural circuit dysregulation.

In addition, Cd and Co were identified as key environmental drivers of PDC through shared pathways of oxidative stress and neuroinflammation. The ubiquitous pollutant Cd consistently associates with PDC, crossing the blood–brain barrier to accumulate in limbic brain regions. It depletes glutathione, generates reactive oxygen species (ROS), and triggers neuroinflammation, leading to disrupted neurotransmitter homeostasis (serotonin, dopamine) and impaired hypothalamic–pituitary–adrenal (HPA) axis function (37, 38). Co’s significant positive association with PDC, evident after full confounder adjustment, is mediated by its pro-inflammatory effects—mitochondrial dysfunction and NF-κB-mediated upregulation of cytokines (TNF-*α*, IL-6)—which sensitize nociceptive pathways and disrupt neuroplasticity (39, 40).

The BKMR model’s identification of cumulative risk from mixed metal exposure underscores the importance of moving beyond single-metal analyses to consider the real-world scenario of co-exposure. The synergistic or additive effects of Cd, Co, Tl, and Tu likely stem from their shared activation of oxidative stress and inflammatory pathways, which converge to disrupt pain and mood regulation. For example, Cd-induced HPA axis dysfunction may enhance the neurotoxicity of Tl, while Co’s pro-inflammatory cytokines may amplify Tu-mediated oxidative damage, creating a multi-faceted assault on the CNS and pain pathways. This mixed-exposure dynamic explains why the cumulative metal burden was a stronger predictor of PDC than individual metals alone, emphasizing the need for public health strategies that address co-exposure to multiple neurotoxic and pro-inflammatory metals.

This study’s strengths include its large population-based sample, comprehensive methodological integration, and adjustment for multiple confounders, which enhance the reliability and generalizability of findings. However, several limitations should be acknowledged. First, the cross-sectional design precludes causal inference. Second, the use of complete-case analysis may introduce selection bias if data are not missing completely at random, though our sensitivity analyses demonstrated consistent results. Third, the assessment of PDC relies on self-reported questionnaires, which may introduce recall bias. Fourth, the analysis relied on data from a single NHANES cycle (2009–2010) in which the comprehensive chronic pain assessment was fielded; although this was necessary to address our research question, it affects the contemporaneity of the findings.

## 5 Conclusion

In conclusion, this study identifies Tl, Tu, Cd, and Co as key environmental metals associated with PDC, demonstrating their non-monotonic and dose-dependent neurotoxic effects through advanced modeling approaches. BKMR revealed synergistic impacts of multi-metal co-exposure on PDC pathogenesis. These findings redefine environmental toxicology paradigms by emphasizing the necessity of holistic exposure assessments and advocating for clinical integration of metal biomonitoring in neuropsychiatric evaluations, ultimately bridging gaps between environmental epidemiology and affective-somatic disorder management.

## Data Availability

Publicly available datasets were analyzed in this study. This data can be found here: https://wwwn.cdc.gov/nchs/nhanes.

## Glossary

NHANES: National Health and Nutrition Examination Survey
Tu: Tungsten
Tl: Thallium
BKMR: Bayesian kernel-machine regression
Be: Beryllium
Co: Cobalt
Mo: Molybdenum
Cd: Cadmium
Sb: Stibium
Cs: Cesium
Ba: Barium
W: Tungsten
Pt: Platinum
Pb: Lead
U: Uranium
LOD: Below-limit-of-detection
BMI: Body mass index
CI: Confidence intervals
OR: Odds ratios
SD: Standard deviation
